# Histological and histochemical effects of Gly-phosate on testicular tissue and function

**Published:** 2012-05

**Authors:** Mazdak Razi, Golamreza Najafi, Sajad Feyzi, Ali Karimi, Simineh Shahmohamadloo, Vahid Nejati

**Affiliations:** 1 *Department of Comparative Histology and Embryology, Faculty of Veterinary Medicine, Urmia University, Urmia, Iran.*; 2 *Department of Anatomy, Faculty of Veterinary Medicine, Urmia University, Urmia, Iran.*; 3 *Departemt of Biology Sciences, Faculty of Basic Sciences, Urmia University, Urmia, Iran.*

**Keywords:** *Abnormal sperm*, *Carbohydrate accumulation*, *Gly-phosate*, *Lipid foci*, *Spermatogenesis*, *Testosterone*, *Testis*

## Abstract

**Background:** In this study we aimed to evaluate the impact of chronic exposure to the Gly-phosate (GP) on rat’s testicular tissue and sperm parameters.

**Objective:** Testicular tissue, morphology of sperms and testosterone level in serum of mature male rats were analyzed.

**Materials and Methods:** Animals were divided into two test and control-sham groups. The test group was subdivided into 4 groups (10, 20, 30 and 40 days GP administrated). Each test group (n=8) received the compound at dose of 125 mg/kg, once a day, orally for 40 days while control-sham group (n=16) received the corn oil (0.2 ml/day).

**Results:** Microscopic analyses revealed increased thickness of tunica albuginea, obvious edema in sub-capsular and interstitial connective tissue, atrophied seminiferous tubules, arrested spermatogenesis, negative tubular differentiation and repopulation indexes, decreased Leydig cells/mm^2^ of interstitial tissue, hypertrophy and cytoplasmic granulation of Leydig cells, elevated death, immature sperm and increased immotile and abnormal sperm percentage. The carbohydrate ratio was reduced in first three layers of the germinal epithelium (GE) cytoplasm. The upper layers of the GE series were manifested with low rate of lipid accumulation in cytoplasm, while the cells which were located in first layers were revealed with higher amount of lipid foci. Hematological investigations showed significant (p<0.05) decreasing of testosterone level in serum.

**Conclusion:** The current data provide inclusive histological feature of chronic exposure against GP with emphasizing on reproductive disorders including histological adverse effect on the testicular tissue, spermatogenesis, sperm viability and abnormality which potentially can cause infertility.

## Introduction

Organophosphorus compounds (OPC) could be successfully applied to agriculture. Because of their chemistry these compounds are a subject of increasing interest. A great number of new agrochemicals with different chemical structures and various properties have been synthesized recently (1). OPCs exert inhibition on catalytic site of the enzymes (2). Most of the OPCs have been detected in environment as the contaminants in the drinking water and in the form of residues in food stuffs because of their extensive uses in different fields (3). 

The testicular tissue and the spermatogenesis process are highly sensitive toward the physical and chemical agents. Previous studies reported that OPCs have adverse effects on the genital system of different animals and humankind (3-5). The important toxic impact of OPCs is phosphorylation of proteins in different cells of various tissues. According to previous reports the OPCs such as diazinon and malathion are able to lead in: down-regulation in gene encoding proteins which are participating in transcription (BP75), translation (ribosomal protein S5), and mitochondrial function of cytochrome oxidase subunits I and III (6, 7). 

These compounds are able to influence the testes, for example decreasing the testicular weight gain and spermatogenesis process (8, 9). Some studies showed that any exposure to environmental toxic compounds such as OPCs results in cancers and/or decreased reproductive functions with interfering to gonadothropic hormones surge and also with delaying the genital systems sexual maturity (10-12). The cytoplasmic carbohydrates (mainly glucose) are the preliminary origin to supply required energy to the most of biochemical activities such as mitosis and miosis. Any disruption in carbohydrates metabolism and/or transport through germinal epithelium is able to influence their mitotic and biological activities, which in turn can lead to spermatogenesis arrest in seminiferous tubules (13, 14). Chemical changes in sperm nuclear proteins (protamine), which pack DNA during the last steps of spermatogenesis, is part of the cause to male reproductive toxicity after exposing to different OPCs (15, 16). However there is little information about the effect of Gly-phosate (GP) as an OPC on male testicular system, testosterone and sperm parameters. 

Thus the aim of this study was to investigate the effect of GP on male gonad including: the impact of GP on germinal cells biochemical alterations, such as intracytoplasmic carbohydrate and lipids ratios, any probable changes in spermatogenesis process, tubular differentiation index, repopulation index and different sperm parameters such as sperm count, motility and abnormality in rats as laboratory models.

## Materials and methods

**Animals**

In total 48 mature male Wistar rats, 8 weeks olds and weighting 200±140 g were used in order to perform this interventional- experimental study. The rats were purchased from the Animal Resources Center of Faculty of Veterinary Medicine, Urmia University, Iran and they were acclimatized in an environmentally controlled room (temperature, 20-23°C, and 12h light/12h dark). Food and water were given *ad libitum*. In this study all experiments which conducted on animals were in accordance with the guidance of ethical committee for research on laboratory animals of Urmia University.

**Experimental design**

Following a week acclimation the animals were assigned into five groups as control-sham (n=16) and test groups (n=8 for each test group). All animals from mentioned groups prior to launching of experiment and after the latest step of the treatment were weighted to evaluate any changes in body weight gain (BWG). 

Animals in the control-sham group received the corn oil (0.2 ml/day) in order to evaluate any effect of gavages on purposed parameters of present study and the rats in the test groups were subdivided into 4 groups based on period of test compound administration. Animals in test groups received GP at dose level of 125 mg /kg, b.w., with oral gavages, once a day for 10, 20, 30 and 40 days. The rats were sampled on days 10, 20, 30 and 40 after dosing. 

**Testicular weight determination**

All rats on days 10, 20, 30 and 40 following anesthesia with ketamine 5% (Iran-Razak), 40 mg/kg, i.p. and xylazine 2% (Trritau, Geramny) 5 mg/kg, i.p. were euthanized by using CO_2_ gas in a special device (Adaco LMa, Iran) and immediately following weighting of total body weight (BW) the testicles were excised free of surrounding tissues and weighed on a basbal scale (Delta Range., Tokyo).

**Histopathological analyses**

One half of the specimens were fixed in 10% formalin fixative for histological investigations and subsequently embedded in paraffin. Sections (5-6 µm) were stained with Iron-Weigert (Pajohesh Asia, Iran) for detection of germinal cells nucleuses in the testis in order to histopathological assessment. All of the specimens were studied by multiple magnifications (400X and 1000X). For the quantification of cells and their dimensions we used 100µm morphometrical lens–device (Olympus Co., Germany). The dimensions were expressed in µm. 

**Immune cells infiltration**

In order to evaluate the mono nuclear immune cells infiltration the number of these cells counted per one mm^2^ of the interstitial connective tissue by using morphometrical lens-device (Olympus Co., Germany). The dimensions were expressed in µm. 

**Histochemical study**

One half of the specimens were freshly cut with frozen section (Bright instrument Co LTD, England) and in order to analyze the testicular germinal epithelium carbohydrate ratio, periodic acid shiff (PAS) special staining technique (Pajohesh Asia, Iran) was conducted on specimens. Furthermore the Sudan-Black B (SB) staining (Pajohesh Asia, Iran) was performed to evaluate the rate of lipid foci supplement in germinal epithelium of the both test and control-sham animals.

**Epididymal sperm content, quantitative sperm mortality and morphology**

Epididymides were separated carefully from the testicle under a 10 time magnification provided by Stereo Zoom Microscope (TL_2_, Olympus Co., Tokyo). The left epididymis was divided into three segment; head, body and tail. The epididymal tail was trimmed and minced in 5 ml ham’s F10 medium (sigma co) for 30 min, 6% CO_2_, 36.5^o^C in CO_2_ culture device (LEEC, England). After 30 min the epididymis was separated from the medium, 10 drops were used for analyzing the percentage of sperm viability for each sample. 

Sperm with stained cytoplasm in head piece were considered as death sperm. For this regard eosin-negrosin staining technique was conducted; moreover the sperm with any cytoplasmic droplets in mentioned pieces were marked as immature sperm. The proportion of death spermatozoa was determined by counting 100 squares morphometry devise (Germany) in randomly selected field from 10 smeared slides for each case and mentioned in percentage (16,17). 

**Total epididymal sperm count and motility**

The cauda epididymis sperm reserves were determined using the standard hemocytometric method using the improved Neubauer (Deep 1/10 mm, LABART, Germany) as described by Pant and Srivastava (18) and sperm motility was analyzed with microscope (Olympus IX70., Tokyo) at 10 fields and reported as mean of motile sperm according to WHO method (19).

**Tubular differentiation index (TDI) determination**

To estimate the TDI, the percentage of the STs that were showing more than three layers of differentiated germinal cells from spermatogonia type A, 200 sections (6µm) were prepared and the STs which showed more than three layers considered as TDI positive (13). 

**Repopulation index (RI) calculation**

To determine the RI, the ratio of active spermatogonia (spermatogonia type B with light nucleus in Iron-Weigert staining technique) to inactive spermatogonia (spermatogonia type A with dark nucleus in Iron-Weigert staining technique), in STs was calculated in 200 prepared sections as mentioned earlier (13).

**Serum sampling and hormonal analysis**

After days 10, 20, 30, 40 the blood samples from corresponding animals were collected directly from the heart and the serum samples separated by centrifugation (Hittech, EBA III, Japan). The collected serum samples were subjected to hormonal analysis. The principle of testosterone level measurement in serum was conducted with Radioimmunoassay method by using special kit for rat (WHO/Sigma Asso-RFGC-78/549). The limit of detection (LOD) was 0.12ng/ml for testosterone. The intra-assay and inter-assay coefficients variances for progesterone was found 4.8 (for 10 times) and 9.9 (for 10 times) respectively.

**Statistical analysis**

Statistical analyses were performed on all data using two-way ANOVA followed by a Bonferroni test, using GraphPad Prism 4.00, GraphPad software. All values were expressed as the mean±SD. p<0.05 was considered to be statistically significant.

## Results

**Clinical observations**

GP administration reduced food and water consumption. At the end of treatment period, it came clear that exposing of animals to GP, exerted significant effect on BWG. Corn oil did not exert any significant effect on BWG in control-sham group, while administration of GP reduced BWG in the test group. 

By the time the testicles were decreased in size and weight in GP administrated rats (Table I). Also all animals in the test groups were observed with decreased movement, staggering gait, occasional trembling, diarrhea and spasms 30 days after dosing.

**Histopathological analysis**

Histological observations revealed that the tunica albuginea was increased in thickness on day 40 after dosing of GP. The control-sham group showed normal tunica thickness. Sub-capsular and interstitial connective tissues were manifested with remarkable edema in all of the GP-consumed rats. This situation was advanced by the time. 

**Figure 1:**
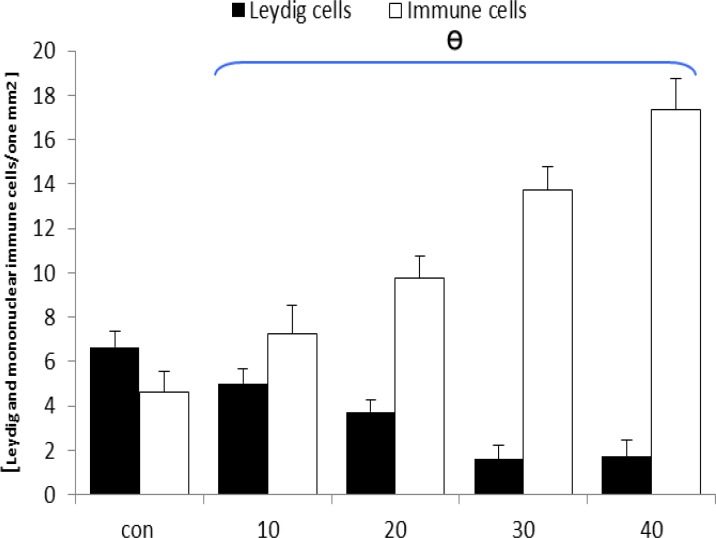
Mean average of the mononuclear immune and Leydig cells per one mm^2^ of the interstitial connective tissue in one cross section from testes, ɵ represents significant differences between all test groups between each other and with control-sham group. p<0.05 was considered as significant differences

After 20 days, a considerable vasodilatation associated with remarkable thrombosis demonstrated in both right and left testicles of the animals in GP-exposed groups. Infiltration of the mono-nuclear immune cells was increased by the time in one mm^2^ of the interstitial connective tissue in GP-inducted cases. This situation was developed by the time in all test animals. After day 30, Leydig cells were seen with a significant decrease in number per one mm^2^ of the interstitial connective tissue (Figure 1). These cells were demonstrated with considerable hypertrophy and cytoplasmic granulation in GP-induced rats. 

**Figure 2:**
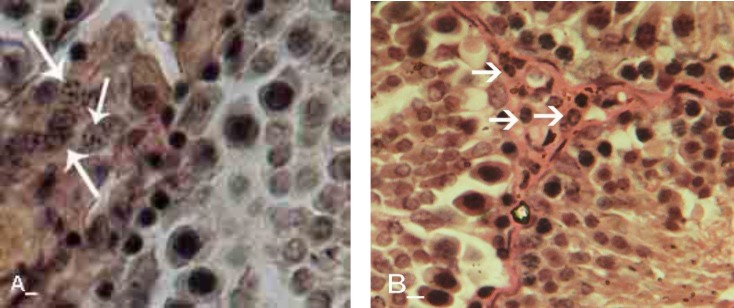
Histological architecture of the testis in test groups, the Leydig cells are presented with cytoplasmic granulation and hypethrophic appearance in aggregated form. (B) Histological architecture of the testis in control-sham group, the Leydig cells are showed with normal appearance, Iron-Weigert staining technique, (400X).

No histological changes were observed in control-sham group (Figure 2 A, B). Light microscopic analyses showed that, the seminiferous tubules (STs) were severely atrophied following GP consumption. After day 20, approximately 90% of the STs in GP-dosed animals were revealed with a significant decrease in cell layers of germinal epithelium (negative TDI index). 

**Figure 3:**
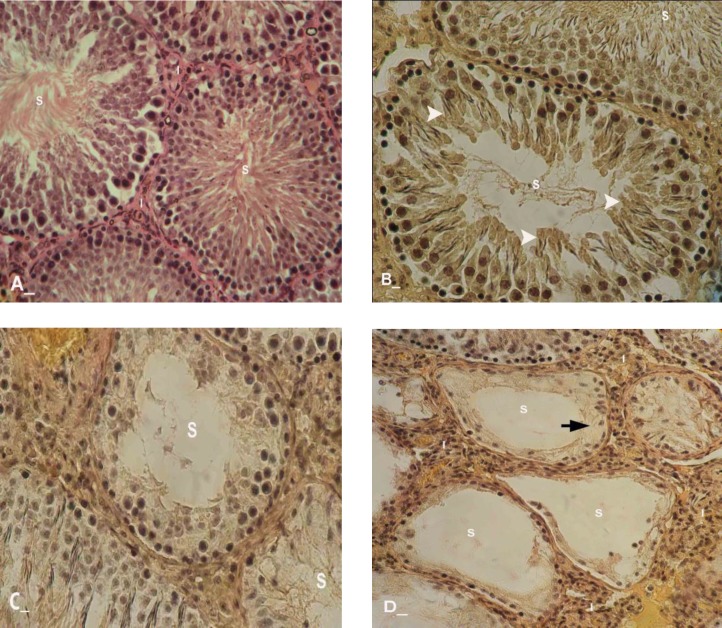
Cross sections from testis; (A) Control-sham, I; indicating interstitial connective tissue, (B) GP-induced group after 20 days, S; with depletion of the seminiferous tubule from spermatozoids, White arrows; indicating spermyogenesis from previous cycle, (C) GP-administrated group after 30 days, T; presenting vascular thrombosis and vasodilatation in interstitial and peri-vasular region, (D) GP- received group after 40 days, I; indicating high immune cells infiltration with completely depleted seminiferous tubule, Black arrow; presenting arrested spermatogenesis, Iron-Weigert staining, (400X, A, B, C and D).

**Figure 4:**
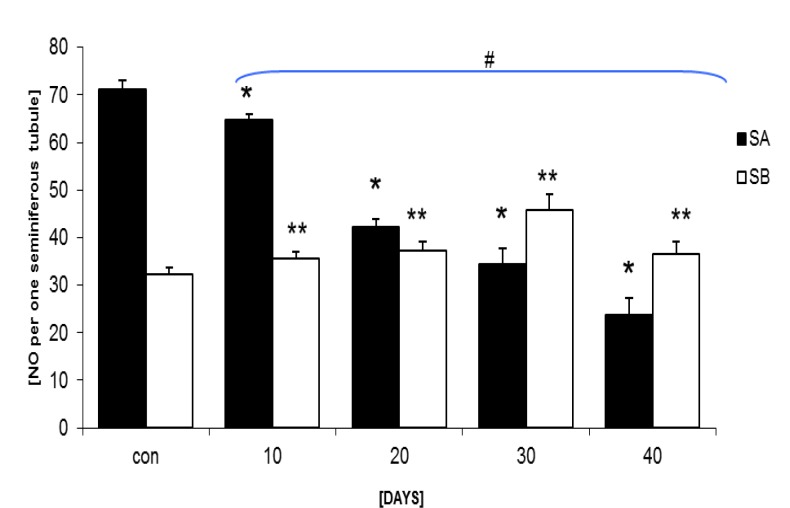
Mean average of spermatogonia type A (S.A) and spermatogonia B (S.B) in one cross section from testes seminiferous tubules, Stars are indicating significant differences between test groups and # representing significant difference between all test groups with control-sham group. p<0.05 was considered as significant differences

No histological changes were observed in control-sham group. Special staining of Iron-Weigert revealed negative RI in GP-exposed rats which characterized with an increased percentage of type B spermatogonia (active cells) to type A (inactive cells). The RI was negative in all of the animals in the test groups, while this situation revealed weakly improved on day 40 after dosing of GP in comparison to other test animals (Figure 3 and 4). 20 days after GP consumption, increased spaces between germinal cells of the STs were revealed. Also the association between Sertoli and germinal cells was disrupted time dependently. No histological changes were observed in control-sham.

**Histochemical observations**

Histochemical analyses of the test cases manifested that the cells in three first layers of spermatogenesis cell series were stained faintly with PAS and the carbohydrate ratio was severely decreased in these cells cytoplasm. The thickness of the basement membrane increased remarkably in test group and this situation was developed by the time. SB staining of the specimens in GP induced rats showed considerable cytoplasmic lipid accumulation in spermatogonia and spermatocytes type I and II cells series. 

**Figure 5:**
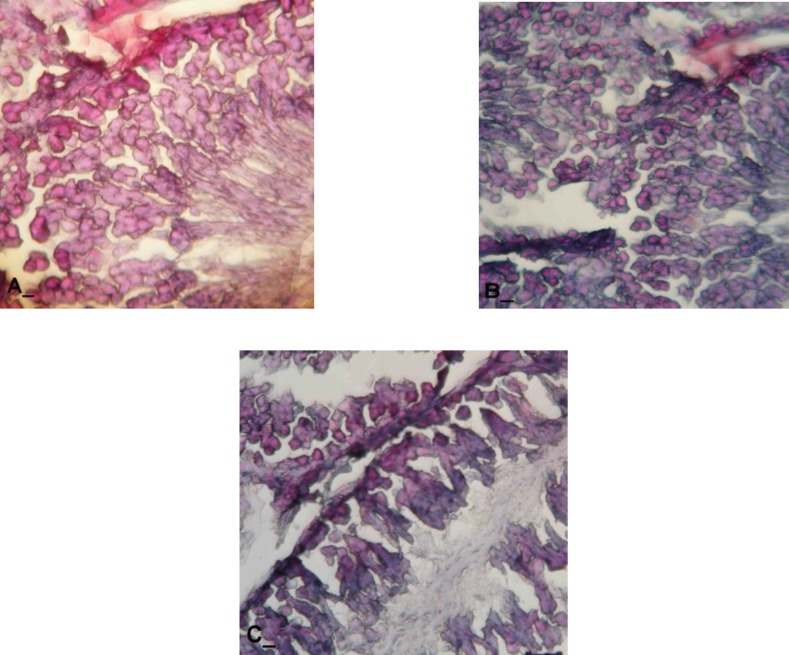
Cross sections from testis, (A) control group, note spermatogenesis cell series in the first three cell layers with unstained SB cytoplasm and the cells in spermayogenesis series which are presented with dark SB positive cytoplasm, (B) GP-induced rats after 30 days, note the cell in first layers of spermatogenesis cell series which are faintly stained with SB and (C) GP-induced group after 40 days, with dark lipid foci in first three layers of spermatogenesis cell series, Sudan Black B staining technique (400X A, B, C)

Spermatids which were adjacent to Sertoli cells and the cells in the upper layers of GE cell series were revealed with lower reacted sites for SB staining. Moreover the Sertoli cells were manifested with darkly SB stained cytoplasm (Figure 5). The animals in control-sham group showed normal cytoplasmic carbohydrate supplement in nearly all of the cells which were participated in spermatogenesis process. 

**Figure 6:**
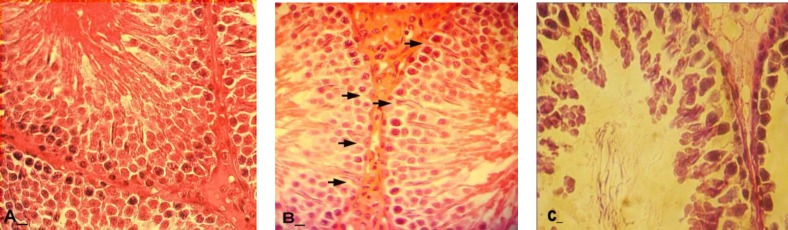
Cross sections from testis, (A) control group, note the Sertoli cells and first three cell layers with dark red stained cytoplasm, (B) GP-induced group after 30 days, note the Sertoli cells with negative PAS reaction (arrows) and the cells in the spermatogenesis cell series with faint PAS stained cytoplasm, (C) GP-induced group after 40 days, completely depleted ST and all cells show negative PAS reaction, PAS staining technique, 400X

Accordingly the Sertoli cells were manifested with well PAS stained cytoplasm showing normal carbohydrate supplement. Also the cells in spermiogenesis process were observed with normal lipid foci accumulation with low reaction to PAS (Figure 6). 

**Sperm morphology and count**

**Figure 7:**
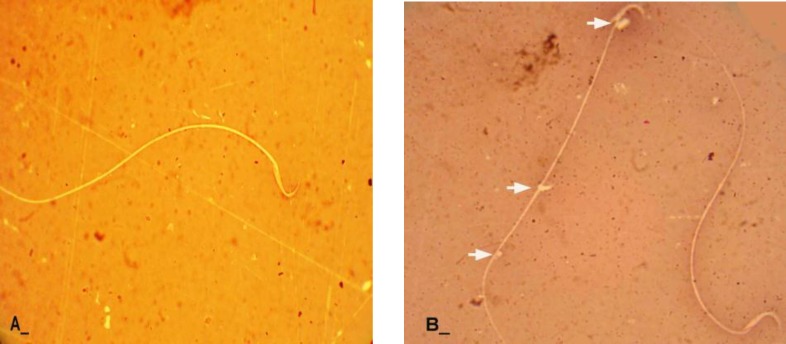
Light microscopic architecture from sperms, (A) normal sperm with unstained cytoplasm (B) immature sperm, (*arrows*) representing cytoplasmic droplet, note the right hand side sperm with stained cytoplasm characteristic as death sperm, Eosin Negrosin staining technique, (400X A, B, C).

**Figure 8:**
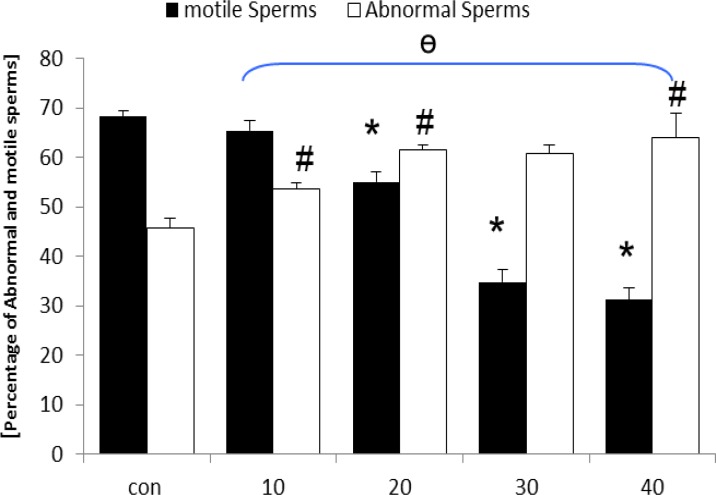
Mean average of abnormal and motile sperms content in control-sham and GP-induced rats, * and # are indicating significant differences between marked test groups with each other and ɵ presents significant difference between all test groups with control-sham group. p<0.05 was considered as significant differences

Light microscopic investigations using special staining of eosin-negrosin revealed increased abnormal sperm volume with decreased sperm viability in the test group in comparison to the control-sham rats (Figure 7). Sperm motility was decreased by the time in all of the test groups in comparison to the control-sham rats (Figure 8). Sperm count analyses showed declined sperm volume in number (Table I). 

**Testosterone measurement**

Serum analyses showed that long usage of GP caused testosterone decline in GP-dosed groups. 

**Figure 9:**
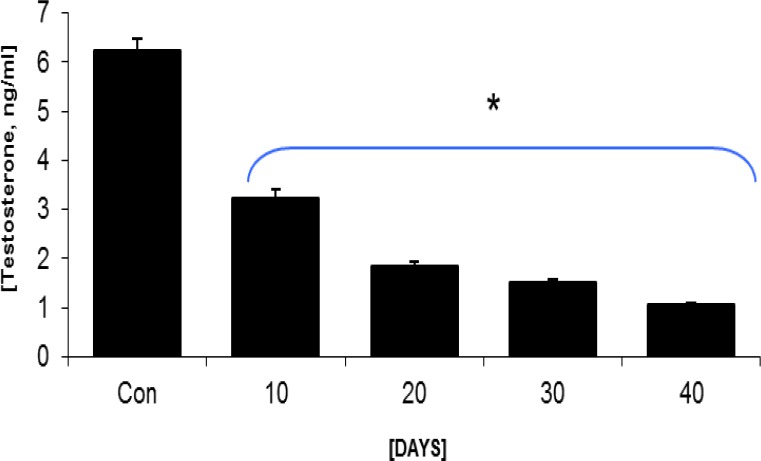
Mean average of testosterone level in control-sham and test groups, * is indicating significant differences between all test groups and between all test groups with control-sham group, p<0.05 was considered as significant differences

Accordingly the cases in GP groups manifested with remarkable decreased testosterone level in the blood circulation during 40 days (Figure 9).

**Table I:** Mean average of body weight gain (BWD), testicular weight gain (TWG) and sperm count in different groups, stars are indicating significant differences (p≤0.05) between all data in different test groups with control-sham group

Groups	BWG (gr)	TWG (gr)	Sperm NO×10^6^
Control-sham	259.66±1.52	12.63±0.44	50.25±4.06
GP-induced, 10 days	205.14±4.22^*a^	11.59±0.73*	43.62±3.24^*^
GP-induced, 20 days	198.14±2.41^*a^	11.03±0.74*	34.12±1.80^*^
GP-induced, 30 days	186.57±1.81^*a^	9.88±0.40^*^	28.37±3.11^*^
GP-induced 40 Days	172.42±1.51^*a^	9.52±0.24^*^	16.62±1.18^*^

Letters in same column are indicating significant differences (p≤0.05) between data in test groups with each other, all data are presented in Mean±SD

## Discussion

In this study we found that GP induces detrimental impact on male testicular tissue such as STs Degeneration, spermatogenesis impairment, imbalance carbohydrates and lipids distribution in germinal epithelium and increased sperm abnormalities. Moreover this compound is able to induce hormonal impairments. 

GP (N-phosphonomethyl glycine) is a non-selective systemic herbicide which is categorized in OPCs groups that is used to kill weeds, especially perennials and broadcast or used in the cut-stump treatment as a forestry herbicide (17). Previous studies indicated that OPCs have adverse effects on male and female genital systems by changing of mass hormonal levels and also by affecting the biochemical functions of the cells in the genital tract (4, 10, 12). Many pesticides decrease the weight of the testes and prolonged-exposure to OPCs diminished food and water intake in treated mice (20, 22). 

Thus in this study we aimed to show how GP-exposure could exert pathological impact on the testes tissue in detail. Very early finding of the present study was that after day 10, GP resulted in decreased testicular weight in the test groups in comparison to the control-sham cases and also animals in the test groups were manifested with decreased BWG. Several reports have showed that OPCs-treatment may inhibit spermatogenesis and make destruction of all kind of GE series (23, 24). It is known that OPCs cause GE disruption that in turn this situation can lead to severe ST atrophy (25). In corroboration with previous reports, we observed severe STs atrophy in the test groups which was followed by elevated interstitial spaces. This situation was advanced remarkably after 30 days. 

According to last investigations spermatogenesis disruption might be due to increases in serum level of LH which is detrimental to affect the germinal cells (26, 27). In the present study the number of primary spermatocytes and round spermatids per STs was decreased by the time and also long usage of the GP caused negative TDI and RI in more than 90% of STs. According to previous reports the OPCs are able to inhibit the non-specific esterase activity in Leydig cells, which in turn it can result in decreasing of testosterone production (28, 6). Testosterone, through modulation of P-mod-S in the peri-tubular cells, could affect Sertoli cell function (29-31). Any functional damage in Sertoli cells can lead to germinal cells degeneration and dissociation. Our hematological analyses demonstrated that long term usage of the GP resulted in declined testosterone level in GP-exposed animals. 

Our results indicated that the Sertoli cells were severely degenerated and the junction between germinal epithelium cells and Sertoli cells was disrupted. Also the number of Leydig cells was decreased by the time. Therefore, it would be logical conclusion that GP-exposure resulted in Leydig cells degeneration, which it resulted in remarkable serum level testosterone reduction and ultimately caused Sertoli cells dysfunction. Consequently, Sertoli cells dysfunction in turn could be able to result in germinal cells degeneration and dissociation (10). Further histological investigation was conducted to explain, how GP can cause this pathogenesis. 

We used the special method of the SB to evaluate the lipid accumulation in the cytoplasm of the GE in STs and interstitial connective tissue of the testes. Comparing the reaction density between the control-sham and GP-received groups indicated that GP caused a significant increase of spermatogonia cells lipid accumulation in contrast to control-sham group which mainly spermatids showed relatively high density of lipid foci. According to these histochemical results GP-exposed rats showed significantly increased lipid supplement in the germinal epithelium of the STs and also in the interstitial connective tissue cells. 

To illustrate how GP causes enhancement of lipid accumulation in mentioned cells, it is interested to be noted the fact that lipid supplementation in the Sertoli cells differs depending on various conditions. When these cells phagocyte residual bodies or degenerated cells the ratio of the lipids increases in the cytoplasm of those cells (32). Therefore, this situation suggests that due to GP-exposure the rate of degenerated cells are increased as we showed already and in turn it resulted to more actively phagocytosis by Sertoli cells, which are represented by densely SB staining. Another reason for lipid accumulation and consequently dominant Sudan Black-B staining in GP-exposed animals may be an interruption in glucose metabolism or transport as a main source of energy to synthesis of the proteins in the germinal cells. 

Since glucose transporter are the main transport pathway of glucose to the STs and spermatogonia (33), thus any degeneration event made by GP could result in interruption in glucose metabolism and ultimately lipid accumulation in the cytoplasm of the cells specially those close to lumen of the STs. According to previous reports which support our results, the testosterone reduction following long time exposure to OPCs leads to severe sperm reduction (28, 29). 

There are other theories which are indicating that the OPCs are able to stimulate lipid peroxidation, changes in the actions of antioxidant agents, DNA damage, and oxidative stresses which can result in cellular death (34, 35). Previous reports indicated that the reactive oxygen species (ROS) production correlates negatively with the sperm quality (13, 37). “A variety of components in male genital system are capable of generating ROS, including: morphologically abnormal spermatozoa, precursor germ cells, leukocytes and at last degenerated cells in spermatogenesis series” (38). There are several reports indicating that OPCs can cause oxidative stresses by increasing germinal cells precursor, degeneration and by abnormal sperm production (35, 39). 

There are many supportive results in the present study including; elevated abnormal sperm content with different characteristics such as; elongated head, pyriform (pear shaped) head, bent and cytoplasmic droplets, increased infiltration of immune-mono-nuclear cells/mm^2 ^of the interstitial connective tissue, degenerated germinal cells in GP-exposed groups which are indicating the probable major role of imbalanced oxidative stress in generating of various disorders.

On the other hand, spermatozoa are highly susceptible to damage by excessive concentrations of ROS due to the high content of polyunsaturated fatty acids within their plasma membrane (37-40). Whenever the ROS concentration elevates, in turn it can lead to remarkable increasing in lipid peroxidation. The lipid peroxidation destroys the structure of lipid matrix in the membranes of spermatozoa, and it is associated with loss of motility (40-42). In the present study GP-exposure decreased sperm motility and viability. Accordingly by the time the rats in the test groups were manifested with high sperm mortality and low sperm volume.

## Conclusion

Based on our results the male reproductive tract can be considered as a target for GP. It can result in histological damages on testicular tissue, increasing sperm mortality, abnormality, decreasing sperm motility and viability. Thus, this compound may cause infertility problems in chronically-induced cases.
